# On the positive correlation between education and fertility intentions in Europe: Individual- and country-level evidence^☆^

**DOI:** 10.1016/j.alcr.2014.01.005

**Published:** 2014-09

**Authors:** Maria Rita Testa

**Affiliations:** Wittgenstein Centre for Demography and Global Human Capital (IIASA, VID/ÖAW, WU), Vienna Institute of Demography, Austrian Academy of Sciences, Wohllebengasse 12-14, 6th Floor, 1040 Vienna, Austria; Vienna University of Economics and Business, Welthandelsplatz 1, Building D4, 1020 Vienna, Austria

**Keywords:** Fertility intentions, Reproductive decision-making, Education, Multilevel analysis, Eurobarometer, Europe

## Abstract

Increasing shares of European women are making large investments in their human capital. Whether and to what extent these investments are in conflict with reproductive behaviour are issues that have repercussions for fertility levels. Using two Eurobarometer survey data (2006 and 2011) on individuals clustered in the 27 EU countries, I investigate the relationship between women's education and lifetime fertility intentions. Results suggest that a positive association between women's level of education and lifetime fertility intentions exists at both the individual and country levels, as well as in a micro–macro integrated framework. The main explanation for these findings—which remains to be proven by future research—is that, in institutional contexts allowing highly educated women to have large families, women of reproductive ages are more prone to make investments in both human capital and family size, because these choices are not seen as incompatible alternatives.

## 1 Introduction

Fertility intentions play a central role in explaining contemporary fertility trends: they are among the strongest predictors of subsequent fertility, and operate as key proximate variables in predicting fertility behaviour (Ajzen, 1991; Schoen, Astone, Kim, & Nathanson, 1999).

The complex effect of education on fertility has been widely studied in the literature, and is a highly relevant topic in research on reproductive behaviour (Kohler & Rodgers, 2003). The diffusion of modern contraception has not levelled the socio-economic differentials in completed fertility (Sweet & Rindfuss, 1983), as women who are college graduates still tend to have fewer children than women with high school degrees or lower levels of education (Yang & Morgan, 2003). Fertility intentions are an important channel through which education affects fertility. However, the relationship between fertility intentions and education is not necessarily the same as the relationship between actual fertility and education and little empirical research has been devoted to this issue. Empirical evidence indicates that highly educated people intend to have more children than less educated women (Heiland, Prskawetz, & Sanderson, 2008), but they ultimately have fewer children (Bongaarts, 2001; Quesnel-Vallée & Morgan, 2003). Moreover, highly educated women revise their birth intentions downwards more frequently than less educated women (Iacovou & Tavares, 2011), especially when they are near the end of their fertile years (Liefbroer, 2009).

A positive and statistically significant cross-country correlation between the mean ultimately intended family size (the number of children already born plus the number of children the individual plans to have in the future) and the proportion of highly educated women of reproductive ages (20–45) has been observed in the three cross-sectional rounds of the Eurobaromter (EB) survey conducted in 2001, 2006, and 2011 (Testa & Grilli, 2006; Testa, 2010, 2012a).

It would be particularly valuable to gain more knowledge about the impact of education on fertility decision-making in Europe given that in many European countries the share of highly educated women has been increasing over time while fertility has been declining.

The objective of the study reported here is to estimate how women's level of education influences women's lifetime fertility intentions through both individual- and aggregate-level effects and to illustrate the responsiveness of such relationship to different demographic and socio-economic characteristics.

The study includes 27 countries of the European Union in which the two Eurobarometer surveys were undertaken, at the beginning of 2006 and 2011, respectively. I focus on lifetime fertility intentions, i.e., the number of children planned for the whole reproductive career, and estimate models for childless, parents with one child, and parents with two children separately because of the fundamentally different process involved in the decision to have a first, a second, or a higher birth order child.

The research aim is pursued by answering the following research questions: (1) Are women's educational levels and intended family sizes positively correlated? (2) What factors are responsible for this positive correlation? (3) How does this correlation vary from country to country; and, within countries, among women at different parities? (4) Does education at contextual level have an impact on woman's fertility intentions above and beyond that of her own education?

These are important questions to answer for both theory and policy reasons. They matter in terms of theory because they allow us to test the appropriateness of conventional explanatory and predictive models of decision-making about family formation for the target group of highly educated people. They matter in terms of policy because a gap between the desired and the actual family size has been found in European countries (Goldstein, Lutz, & Testa, 2003). This gap is particularly large among highly educated women, who typically have lower actual fertility levels but higher reproductive intentions than their less educated counterparts (Testa, 2012a). A reduction of such a gap is widely considered to be an important goal.

The remainder of the paper is organised as follows. First, I review the relevant literature on fertility and fertility intentions at macro and micro level. Next, I present research hypotheses, data, and methodology. This is followed by a description and interpretation of the main statistical findings and a discussion of possible caveats inherent to the analysis.

## 2 Theoretical framework

### 2.1 Explanations of low fertility

A variety of theories have been developed to explain low fertility. Each of these theories proposes a different approach that emphasises a particular set of determinants.

The most relevant theories for the study of fertility behaviour of highly educated women are the economic and the gender theories because they explicitly consider the effect of women's working career on their childbearing.

The socio-economic explanation for low fertility focuses on the direct and indirect opportunity costs of having children (Becker, 1981a,b). According to this theory, women's increased economic independence, which is achieved through improved education and higher labour force participation, reduces the gains from marriage based on the interdependence of the traditional gender division of labour in the family, and increases the relative costs of childbearing. In other words, it is assumed that women forgo earnings to care for children at home, or that they reduce their work hours.

A second group of theories identify gender systems and gender inequality as the main sources of fertility differentials across countries, and are often used to explain the lowest-low fertility found in southern Mediterranean countries. McDonald (2000) has suggested that very low fertility may be the result of a hiatus between high levels of gender equity in individual-oriented institutions and sustained gender inequity in family-oriented social institutions. While women have, in recent years, had the same opportunities as men in education, and to some extent in the labour market, this has not occurred within the family. Women have become more empowered in their decision-making in relation to both household labour and fertility because their high levels of education allow them to question traditional roles (McDonald, 2006). According to the gender theory, the countries that successfully adapted to the demise of ‘traditional family’ based on marriage and male breadwinner model record higher fertility levels than countries with incomplete gender and family “revolutions” (Esping-Andersen, 2009; McDonald, 2000).

### 2.2 Education and reproductive decision-making

The theory of planned behaviour (TPB) (Ajzen, 1991) posits that intentions are the most proximate determinant of the corresponding behaviour. According to this theory, intentions are formed under the immediate influence of three groups of factors: (a) personal positive and negative attitudes towards the behaviour, i.e., having a child; (b) subjective norms, i.e., perceived social pressure to engage or not to engage in the behaviour; and (c) perceived behavioural control, i.e., the ability to perform the behaviour, which may depend, for example, on the availability of housing, income, or other resources. Billari, Philipov, and Testa (2009), who have applied the general theory to the case of fertility, showed that the transition to parenthood is mainly driven by the existent normative pressure and individual personal attitudes towards childbearing, while perceived behavioural control plays a bigger role in the decision to have a second child. It may be assumed that perceived behavioural control has a positive effect on the fertility intentions of highly educated women (Testa, 2010). The question is whether, and to which extent, the positive effect exerted by the perceived behavioural control might be counterbalanced by a negative effect exerted by the norms and attitudes. Norms contribute substantially to the negative effects of educational enrolment on women's fertility (Billari & Philipov, 2004; Blossfeld & Huinink, 1991; Morgan & Rackin, 2010), which demonstrates the importance of enrolment itself, regardless of the achieved educational level. Reverse causality may also be at stake here: empirical studies have shown that women with advanced degrees have lower completed fertility on the average because women who have one or more children early are more likely to leave (or not enter) long educational tracks and never achieve a high educational level (Cohen, Kravdal, and Keilman, 2011). In the motivational traits-desires-intentions-behaviour theoretical structure (Miller, 1994), individuals go through a sequence of steps that starts with psychological traits, such as childbearing motivations, and are activated by desires, which are in turn translated into intentions. The final outcome of the childbearing decision process is a conception and a fertility event related to it, such as childbirth or an induced or spontaneous abortion. Traits are defined as a disposition to feel, desires are wishes that do not lead to action, and intentions are conscious commitments to act that take into account the perceived desires of significant others, especially of the partner, and other situational factors. Miller (1992) demonstrated that childbearing motivations are negatively associated with educational level because having a high level of education gives women a higher degree of autonomy which in turn promotes activities competitive with childbearing and leads to wishes for fewer children. The exposure to life course paths competitive with childbearing, such as the completion of education, also plays a crucial role in explaining the transition to parenthood (Barber, 2001). The sign of the correlation between women's education and reproductive intentions depends on whether the desires of significant others and the situational constraints considered by highly educated women in their decision-making process counterbalance the negative effects that stem from their increased level of autonomy.

Highly educated women tend to substitute child numbers with child quality (Becker & Lewis, 1973). Since childbearing and childrearing are time-intensive, an increase in wage rates induces a negative substitution effect on the demand for children (Becker, 1965). A woman's income is, therefore, negatively associated with childbearing, as having a higher income level implies that opportunity costs associated with having children are higher. For men, by contrast, the positive income effect tends to dominate, as they spend less time raising children, although the magnitude of these effects will vary across countries and birth parities (Butz & Ward, 1979). Consistent with this view is the hypothesis that the time demands and the values associated with higher-status occupations compete with positive childbearing motivations (Miller, 1992), and induce women in such positions to postpone the birth of their first child in order to achieve an optimal trade-off between human capital investments and career plans (Gustafsson, 2001).

The main mechanisms through which women's educational level is expected to be positively correlated with women's (or couples’) fertility intentions are linked to the income effects postulated in the economic theory and to the gender equality effects envisaged in the gender theory. Highly educated women have higher average earnings that can make a plan for larger family more realistic and affordable; this is especially true because they usually have a partner who is also highly educated and for which the income effect clearly dominates (Becker, 1981a,b). Similarly, highly educated women are more often in gender equal partnerships in which the man contributes substantially to the housework and childcare duties and this can encourage plans for larger families (Mills, Mencarini, Tanturri, & Begall, 2008).

## 3 Research hypotheses

Highly educated women are exposed to life course paths that compete with childbearing, but they do not necessarily plan to have smaller family sizes than less educated women (Hayford, 2009; Heiland et al., 2008; Mills et al., 2008). Some women in high-status occupations may intend to have fewer children from the beginning of their reproductive careers (Friedman, Hechter, and Kanazawa, 1994), while others may later decide to forgo having some of the children they had initially planned to have over the course of their reproductive careers (Iacovou & Tavares, 2011). Better educated women are more prone to postpone having children than less educated women (Heaton, Jacobson, and Holland, 1999; Schoen et al., 1999), and, consequently, they are more likely to have fewer children than they had initially intended. The mechanisms that could account for this are: (1) declining fecundity with age, which may result in involuntary childlessness; (2) repeated postponements, because of competing activities; (3) lack of partner, or partnership instability (Morgan & Rackin, 2010). It is unclear whether and to what extent highly educated women are able to anticipate the negative effects of postponement on their reproductive careers. This ability may be captured by the level of certainty attached to their fertility intentions since uncertainty may be an acknowledgement that delaying childbearing could lead to forgoing having children (Morgan, 1981, 1982). We could expect that after the transition to parenthood the limited time left out for having additional children is reflected in a higher level of uncertainty attached to the reproductive plans and that after controlling for this uncertainty the intentions of highly educated women become lower than those of the less educated counterparts.

A crucial issue in investigating the relationship between women's human capital and fertility intentions is whether the positive income effect is greater than the negative substitution effect. I focus on three different channels through which the positive effect of the women's increased education on fertility decisions may be strengthened: availability of childcare services, gender equality, and economic conditions.

An important extension to the argument provided by Becker is based on the assumption that women's fertility decisions depend not only on their wages, but also on the availability of external childcare. At the highest level of education, the income effect may be greater than the substitution effect, especially when childcare can be purchased in the market (Del Boca & Pasqua, 2005; Ermisch, 1989).

Cross-sectional studies of differences in the relationship between women's human capital and fertility decisions might reflect the differences across countries in the provision of childcare services. I formulate my first research hypothesis as follows:

HP1 The relationship between women's level of education and lifetime fertility intentions is positive in those countries in which the availability of childcare services offsets the high opportunity costs paid by highly qualified women for having children.

Both of these effects are assumed to be more pronounced after the birth of a first child.

In addition to the income and the substitution effects, a third mechanism linking income to childbearing is the price of time effect, which refers to the ability to combine work and family (Becker, 1981a, 1981b). If gender relations within the couple move in a more egalitarian direction in response to the increased economic opportunities of highly educated women, the lower price of time effect can compensate for the higher substitution effect among highly educated women (Oppenheimer, 1994). The literature has shown that, in egalitarian gender systems, the price of time effect may be reduced for women (Jansen & Liefbroer, 2006; Liefbroer and Corijn, 1999), and that, in countries in which high levels of gender equity in education and the labour market are combined with low levels of equity in the family, fertility is particularly low (McDonald, 2000). I formulate my second research hypothesis as follows:

HP2 The relationship between women's level of education and lifetime fertility intentions is positive in those countries in which egalitarian gender roles in the family and in the market offset the high price of time paid by highly qualified women for having children.

As we saw for hypothesis 1, the effects are assumed to be particularly pronounced after the birth of a first child, when a woman has a better idea of the amount of help with childcare duties she can expect to receive from her partner (Mills et al., 2008).

A positive relationship has been detected between child-timing intention (i.e., the intention to have a child in the next three years) and a country's level of GDP per capita (Testa, 2010): i.e., people living in countries with a high GDP per capita tend to anticipate the birth of a second child. This finding is in line with studies showing a positive link between fertility and economic development (Luci & Thévenon, 2011; Myrskylä, Kohler, & Billari, 2009) and suggests that reproduction and economic development are not necessarily negatively associated. I therefore formulate my third research hypothesis as follows:

HP3 The relationship between women's level of education and lifetime fertility intentions is positive in those countries with a higher level of GDP per capita.

Here I assume that a country's favourable economic conditions may have positive repercussions for fertility, as has been shown in previous studies (Luci & Thévenon, 2011). There could be several mechanisms driving such a relationship: the high levels of GDP per capita are also typically linked with an increased level of well-being and life satisfaction (Testa, 2012a) which may bolster fertility and fertility intentions, especially those of highly educated women.

## 4 Data and methods

### 4.1 The sample

The empirical analysis is based on the Eurobarometer surveys carried out in 2006 and 2011 in the 27 EU countries. In each of these surveys the stratified sampling procedure assures nearly equal probability samples of about 1000 respondents in each of the countries. The sample size allows equally precise estimates for small and large countries, as well as to make comparisons between sub-groups broken down by sex, age, education, marital status, and so on. The surveys used a single uniform questionnaire design, with particular attention being paid to equivalent question wording across languages.

After having pooled together the 2006 and the 2011 EB rounds, the analytical sample consists of 9452 women aged 20–45 who answered the question on fertility intentions: 3332 childless, 2627 with one child, and 3493 with two children. The non-response rate was slightly less than 10%. A missing answer may be symptomatic of certain fertility plans (Morgan, 1981, 1982). However, I simply excluded from the analysis all individuals who did not report any intended family size in order to avoid potential complications given the absence of auxiliary information on this item. The results obtained from the analysis run on the sub-set of valid responses are reliable under the standard “missing at random assumption” (Little & Rubin, 2002).

The models are formally based on two levels: individuals and countries (referred to as “clusters”) as described in Table 1. As is shown in this table, the hierarchical structure is quite unbalanced. This lack of balance is not a problem, as it is efficiently handled by maximum-likelihood methods. The number of clusters and their sizes are sufficient to achieve high levels of power and accuracy of the asymptotic distributions of the estimators (Stegmueller, 2013; Snijders & Bosker, 1999), and thus allow for reliable inferences. Multilevel models assume random sampling at all levels, while our survey design in fact does not use sampling at the country level. Even in such a circumstance, multilevel models could be useful because they allow the explicit inclusion of country-level explanatory variables and country-level residual variation (Hox, vand de Schoot, & Matthijsse, 2012).

For the estimates computation I used the programme gllamm which runs in the statistical package Stata and estimates GLAMMs (Generalised Linear Latent and Mixed Models) by maximum likelihood, i.e., via a maximisation algorithm with adaptive quadrature, assuming Gaussian random effects (Skrondal & Rabe-Hesketh, 2004).

### 4.2 Response variable: lifetime fertility intentions

The response variable, i.e., the intended number of children, was measured through the following item: “*How many more children do you intend to have?*” A range from zero to up to six children was given in the questionnaire as a response option. The prospective item was asked immediately after the question about the number of children already had (“*How many children, if any, have you had?*”) and was clearly intended to provide information about the number of births respondents plan to have over (the rest of) their reproductive careers. Neither of the above-mentioned questions made a distinction between biological and adopted children. Moreover, since pregnancies are not measured in the survey, it cannot be excluded that pregnant women reported the children already conceived at the time of the survey as expected to be born, i.e., in the intended component of their ultimately intended family size.

The response variable was coded as a four-category variable: zero, one, two, and three or more children. Values greater than or equal to three were, in light of their low frequency, collapsed into a single category.

Certainty levels of lifetime fertility intentions were also used. They were measured through the following survey item: “*How certain are you that you will have the number of children that you have just mentioned*?” Response options were: “very sure”, “fairly sure”, “not very sure”, and “not at all sure”. All of the respondents who provided a valid numerical answer other than “0 child” to the question on the number of children they intended to have answered the question about their certainty level.

All the above mentioned variables were measured exactly in the same way in the two EB rounds, 2006 and 2011, which allowed me to run the regression analysis on a pooled dataset.

### 4.3 Explanatory variables

The explanatory variables of the models are as follows: age, enrolment in education, level of education, marital status, employment status, and self-location on the social scale. All of the covariates refer to the time of the interview. Unfortunately, the data do not carry any retrospective information concerning the previous history of respondents, which would have allowed me to estimate the role of biographical trajectories on the process of forming family size intentions in a dynamic framework. The assumption of constancy over time is quite reasonable for some covariates, like, for instance, completed educational level; for the other covariates, I simply assume that they exert an influence as they are measured at the time of the survey, independently on whether the different statuses (marital, employment, social) have been reached since long or short time.

Since the main focus of the analysis is on women, models were restricted to a female sub-sample. EB data from 2006 and 2011 were pooled together, while testing for interaction effects between ‘round 2006’ and all of the other explanatory variables in the models. No significant interactions were detected; hence, interaction terms were not retained in the final models. All the covariates included into the models were measured exactly in the same way in the EB 2006 and 2011, which allowed me to code them exactly in the same way before pooling the data together.

The age of respondents is the only continuous covariate. It was centred on the rounded mean value of 33 years. As all of the other covariates are categorical, they were transformed into suitable dummy variables. Some collapsing of the categories was often needed: in such cases, several alternative collapsing schemes were tried in the model selection process.

The educational level was measured with the following survey question: “*How old were you when you stopped your full-time education?*” and considered as a three-category variable with low (up to 15 years) medium (between 16 and 19) and high (20 years or above) level of education. This code reflects the three education categories as available in the EB survey. A dummy variable indicating whether respondents were still enrolled in education at the time of the survey was also added.

The marital status was coded using four categories: single, married, cohabiting, and separated. The ‘separated’ category included also divorced and widowed people not living with another partner at the time of the survey, while the married category included also remarried people.

The employment status has three categories: employed, unemployed and not active in the labour market.

The country-level explanatory variables of the models are as follows: the gross domestic product (GDP) in purchasing power standards (PPS) as of 2012, taken from the Eurostat database; the share of women in the country with higher levels of education (levels 4, 5, and 6, according to the 1997 ISCED classification), taken from the Labour Force Survey (year 2008); the share of enrolment in formal childcare for pre-school children aged three to five, taken from OECD Family Database (OECD, 2012), and the Gender Empowerment Measure, which is an indicator of gender equality intended to measure women's and men's abilities to participate actively in economic and political life and their decision-making over economic resources, which is computed by the United Nation Development Programme (UNDP, 2007, 2009).

The volume index of GDP per capita in purchasing power standards is expressed in relation to the European Union (EU-28) average, set to equal 100. If the index of a country is higher than 100, this country's level of GDP per capita is higher than the EU average, and vice versa. The basic figures are expressed in PPS; i.e., in a common currency that eliminates the differences in price levels between countries, which allows for more meaningful volume comparisons of GDP between countries.

A description of all the variables used in the models is reported in Table 2.

### 4.4 The micro–macro framework

Multilevel models were run in order to represent the complex causal process underlying the behaviour of individuals living in a social context, and to draw valid inferences regarding the relationships at the relevant hierarchical levels. As is usual in a multilevel setting, the clustering of individuals in countries is a phenomenon of interest, rather than a mere disturbance (Snijders & Bosker, 1999).

**Scheme 1:**
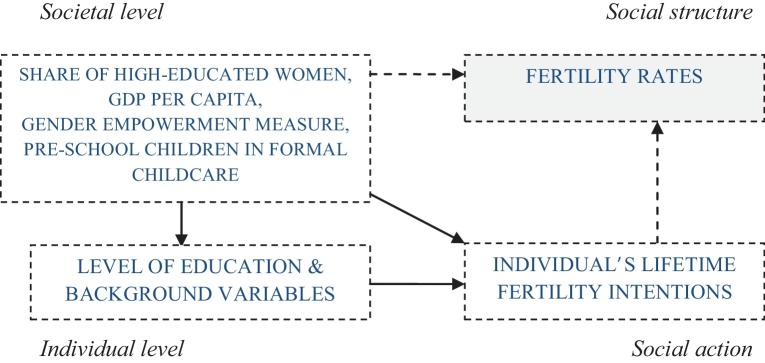
A micro–macro model of fertility. *Source*: inspired by Coleman (1990).

In Scheme 1, freely inspired by Coleman (1990), the multilevel framework is adapted to the study of individual's lifetime reproductive intentions. The box visible at the top right of the scheme is related to fertility rates, which are not investigated in the current analysis, but depend on the relationship explicitly considered in the current study.

A crucial characteristic of the multilevel setting is that the effect of the context on the individual outcome can be estimated after a control for the individual-level characteristics is included in the model (the diagonal line in the scheme).

### 4.5 The model

The multilevel analysis relies on the random intercept version of the proportional odds model for ordinal responses (e.g., Agresti, 2002). All of the models were run separately by parity: zero, one, and two children. As was stated in the rational choice theories approach (Yamaguchi & Ferguson, 1995), fertility intentions may change after each new birth, in line with the concept of a conditional-sequential fertility decision-making process (Namboodiri, 1972). The preference for models stratified by parity over pooled models with parity interactions is reinforced by reasons of parsimony. Models based on a pooled dataset would have required the inclusion of all the interaction terms between each of the parities and all the other demographic and socio-economic explanatory variables, given that the reproductive decision-making is quite different and very sensitive to the number of children already born. A problem arises when there is selection in a parity-specific analysis; i.e., there are unobservable variables that could be correlated with the probability of having a child in parity n, as well as with the probability of intending to have a child of the next order, *n* + 1. The consequence is a biased and inconsistent estimator. This problem is not tackled here because of a lack of adequate longitudinal retrospective information, but the related issue is discussed in the concluding section.

The proportional odds model could be extended to handle partial proportional odds (Williams, 2006), but then the interpretation becomes somewhat tortuous. Since only a few covariates in each model violated such an assumption, and since they did so only slightly, the proportional odds multilevel models were preferred.

## 5 Results

### 5.1 Cross-country differences in ultimately intended family size

A previous study (Testa, 2012b) has provided evidence supporting the consistency of the EB survey data and has also suggested that the ultimately intended family size, as computed from the EB survey data, and the projected cohort fertility (Myrskylä et al., 2012), as computed by using national statistics as a basis for the projection, move in the same direction.

**Fig. 1:**
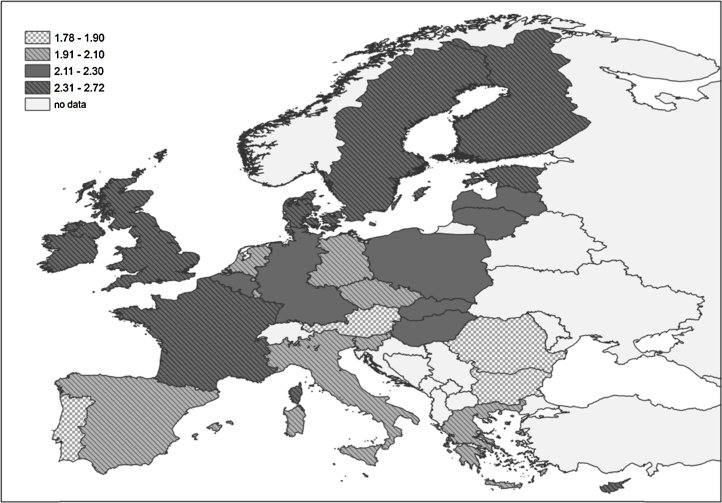
Mean ultimately intended family size in Europe (EU27). Women aged 20–45. *Source*: Eurobarometer data 2011.

Looking at the cross-country differences in the mean ultimately intended family size of women of reproductive ages (20–45) we can spot several clusters of countries with similar values, as shown in Fig. 1.

Going from the lowest to the highest level of ultimately intended family size, the first cluster of countries encompasses Austria, Portugal, Romania and Bulgaria with mean values clearly below the replacement level, ranging between 1.8 and 1.9. The second group includes Italy, Spain, Greece, Slovenia, Malta Czech Republic, the Netherlands, Luxembourg and eastern Germany, with mean values slightly below the replacement level, ranging between 1.9 and 2.1. The third group of countries encompasses western Germany, Belgium, Hungary, Slovakia, Poland, Latvia and Lithuania with mean values at the replacement level, ranging between 2.1 and 2.3. The last group of countries includes Ireland, the United Kingdom, France, Denmark, Sweden, Finland, Estonia, and Cyprus with mean values above 2.3.

This clustering roughly reflects the cross-country family policy differences detected in a recent study (Thévenon, 2011). In the southern European countries, grouped in the first and second clusters with the lowest levels of ultimately intended family size, the family policies are also characterised by limited periods of paid child-related leave, limited provision of childcare services for children under age three, low volumes of cash transfers, but effective tax rates that provide incentives to work and to have a second earner in the household.

The Nordic countries, grouped in the fourth cluster with the highest levels of ultimately intended family size, provide a substantial level of policy support to parents with children under age three, allowing them to easily combine work and family. The forms of support include a long full-time-equivalent period of father-specific leave (around 10 weeks in Sweden, compared to an average of 1.7 weeks across the OECD countries), tax advantages for dual-earner households, and high enrolment rates of children under age three in formal childcare.

The continental European countries, with a mean ultimately intended family size at EU-27 average levels, are mainly grouped in the second and third cluster; the only exceptions are Austria with a lower level and France with a higher level of ultimately intended family size. In these countries the policies are characterised by a generous level of support, which is, however, not targeted at facilitating the balancing of work and family. The level of spending on families with small children is rather high, but the support is aimed at compensating families for the costs of raising children. The taxation system does not encourage the labour market participation of both parents, as the period of leave entitlement is rather long (with the exception of the Netherlands), and the enrolment rates of children under age three in formal care is low; the rates are actually higher in Belgium, France, and Luxembourg; and are lower in the Netherlands, Germany, and Austria.

In the eastern European countries, the policies are rather heterogeneous, with Hungary having the most comprehensive level support for parents with young children. This heterogeneity is consistent with the fact that these countries are present in each of the four clusters outlined above.

A similar clustering of countries was obtained by considering the ultimately intended family size of highly educated women. Only eight countries were listened in a different cluster: Italy, Slovakia, Slovenia and Malta, which were in the adjacent cluster with higher UIFS values, and the United Kingdom, Denmark, Cyprus and Latvia which were in the adjacent cluster with lower UIFS.

### 5.2 The relationship between education and lifetime fertility intentions

**Fig. 2:**
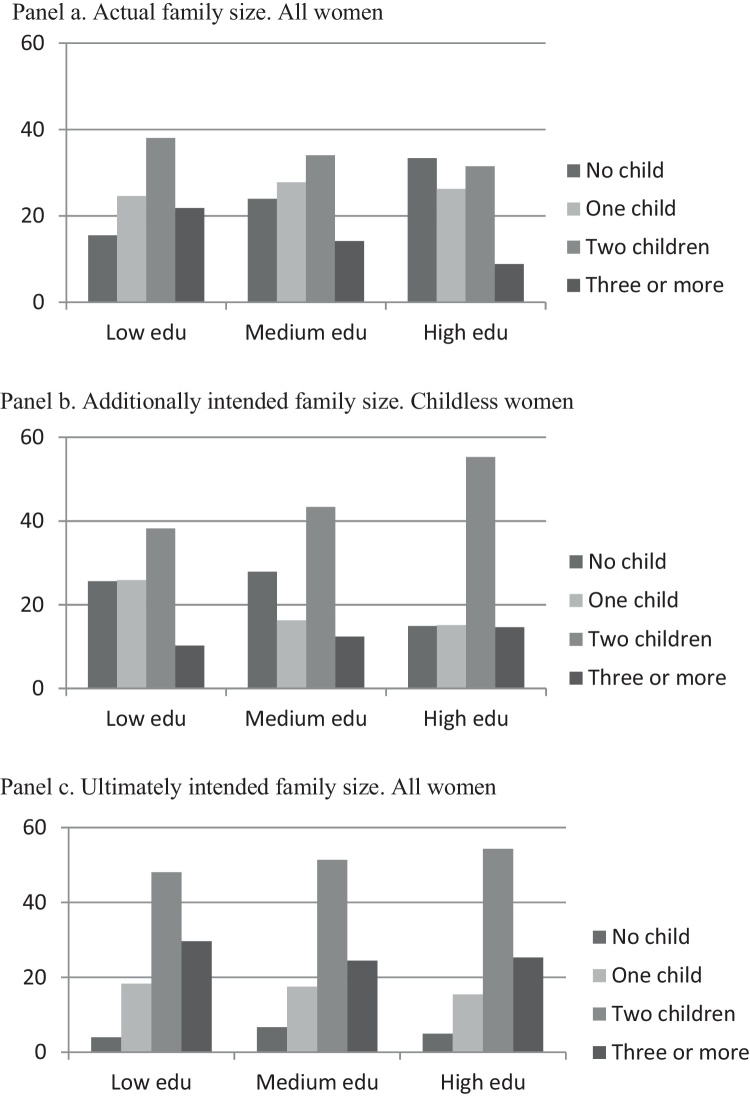
Distribution of women aged 20–45 by actual, additionally, and ultimately intended family size and educational levels. EB 2011.

Looking at the parity distribution of women by level of education in the EU27 as a whole, it is evident that highly educated women are under-represented in the high parities of three or above, but they are over-represented in the lower parities of zero and one, if the actual number of children is considered (Fig. 2, panel a); while they are over-represented in the high parities if the ultimately intended number of children (Fig. 2, panel c) or the additionally intended number of children for the childless sub-sample (Fig. 2, panel b) are considered. These differences are related to the different timing of childbearing adopted by highly educated women and less educated ones, with the former usually delaying family formation longer than the latter. The distribution of women by actual family size also suggests that a bipolarisation process might be behind the reproductive choices of women with high levels of education, in which they more frequently select the “no child” or “two children” option than the “one child” option (Fig. 2, panel a). The two-child family was the most preferred family size of the majority of the respondents in all the three education categories (Fig. 2, panel b and c) while the two-child family was as frequent as the no-child family among the highly educated women (Fig. 2, panel a).

Indeed, in two out of three EU countries, the distribution of highly educated women by the actual number of children showed a higher concentration at parities zero and two than at parity one with the eastern European countries being the main exceptions. However, an analogous bipolarisation was not observed for the lifetime fertility preferences with the only exception of two countries, the Netherlands and the United Kingdom, where having one child was a very uncommon preference (8% and 4% of highly educated women aged 20–45 preferred this option) (Table 3).

Moving on to the analysis of the mean values, it can be noticed that women with high levels of education have a smaller mean actual family size but a larger mean additionally intended family size than their less educated counterparts in most of the EU countries (Table 4).

In 15 of the 27 countries (namely: Latvia, Romania, Bulgaria, the Czech Republic, Hungary, Cyprus, Greece, Portugal, Finland, France, the United Kingdom, Denmark, Austria, Luxembourg, and the Netherlands), the mean ultimately intended family size was higher for the women with low to medium levels of education than it was for the highly educated women. In five of the 27 EU countries (Poland, Lithuania, Slovenia, Germany and Spain), the mean ultimately intended family size did not substantially differ by educational level. In another seven EU countries (Ireland, Sweden, Estonia, Belgium, Slovakia, Malta, and Italy), the mean ultimately intended family size was greater among highly educated women than among less educated women. In this group of countries, the smaller actual family size of highly educated women relative to less educated women was more than compensated for by the larger number of intended children. The only exception was Italy, where both the mean actual and the mean intended family size were higher among women with high levels of education than among women with low to medium education levels. Using the three categories of low, medium, and high levels of education separately, it appeared that less educated and highly educated women had higher mean values than women with medium levels of education in several countries. Here, for the sake of simplicity and because of the limited size of some national samples, the results for women with medium-low and high levels of education are described.

**Fig. 3:**
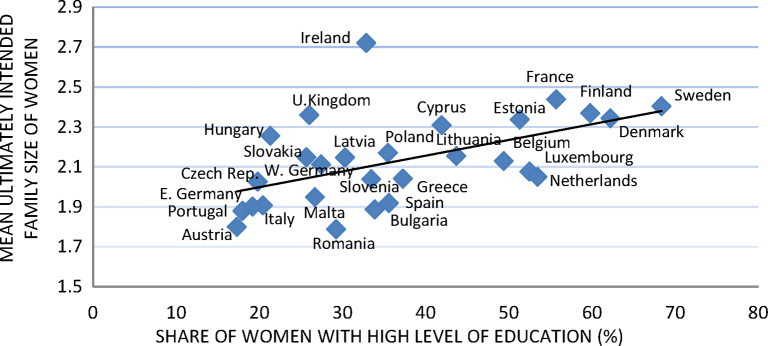
Cross-country correlation between women's mean ultimately intended family size and the share of highly educated women. Ages 20–45. *Note*: Pearson's correlation coefficient is equal to 0.5 and statistically significant. *Source*: Author's elaborations on EB 2011.

The cross-country bivariate correlation between education and lifetime fertility intentions was found to be positive: the countries with a high share of highly educated women of reproductive ages were also the countries in which women of reproductive ages tended to have higher mean ultimately intended family sizes (Fig. 3). The Pearson's correlation coefficient was equal to 0.5 and statistically significant. The correlation was very robust, i.e., it did not change when the analysis was restricted to either childless women or women with only one child, when the Scandinavian countries were excluded, and when the mean additionally intended family size was weighted with the certainty levels of intentions. Looking at the additionally intended family size by parity is crucial because intentions may be higher among childless and highly educated women than among childless and low to medium educated ones just because the former postpone the start of a family more often (or to a greater extent) than the latter. Isolating the Scandinavian countries is important because recent research has shown that in these countries the educational gradient of fertility has been reversed (see, for example, Kravdal & Rindfuss, 2008). Eventually, considering certainty levels of intentions is relevant because uncertainty may well be higher among highly educated women, if we accept the interpretation seeing it as a reflection in people's mind that delayed childbearing could lead to childbearing foregone (Morgan, 1982).

**Fig. 4:**
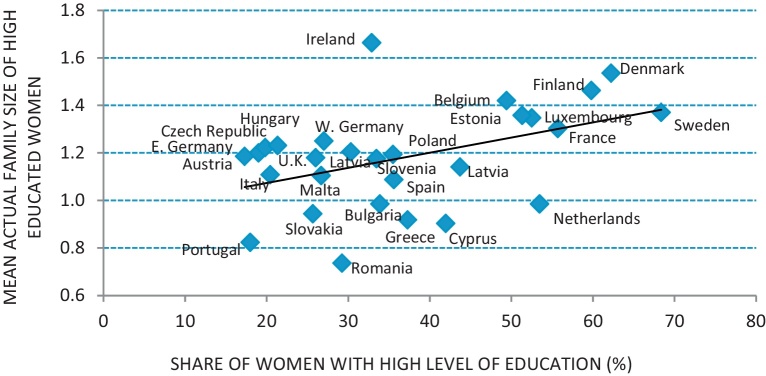
Cross-country correlation between the mean actual family size of highly educated women and the share of highly educated women. Ages 20–45. *Note*: Pearson's correlation coefficient is equal to 0.5 and statistically significant. *Source*: Author's elaborations on EB 2011.

Interestingly, the scatter plot between the share of highly educated women and the mean actual family size of the highly educated women (Fig. 4) roughly resembles the scatter plot showing the association between the share of highly educated women and the mean ultimately intended family size (Fig. 3). This result points out that countries in which women make greater investments in human capital are also those in which highly educated women have larger families.

### 5.3 Results of the ordinal regression models with random intercept

In Table 5, the estimates of the ordinal regression models with a random intercept for the additionally intended number of children are reported. The models were run on the pooled female dataset of EB 2006 and EB 2011 and separately by parity zero, one, and two. Only the additionally intended children were considered in the response variable, to avoid problems of reverse causality which we would have faced by explaining events occurred already in the past (i.e., children already born) with characteristics measured only at the time of the survey (all the explanatory variables are measured at the time of the survey). Explanatory variables have been included gradually in the analysis: model I is the empty one, model II includes only the individual-level variables, and model III includes both the individual- and country-level variables.

As the table shows (Models II), at the individual-level the additionally intended family size is negatively associated with age (for all the three parities) and with the status of being inactive (only at parity zero) and single or separated (at parity one); by contrast, it is positively associated with a high level of education (for all the three parities), with enrolment in education (parity zero and one), and with a high self-positioning on the social scale (for all the three parities). Moreover, there is a positive and statistical significant effect of ‘year 2006′ in the model run on parity one, which suggests a decrease in the intended family size across the two EB waves, 2006 and 2011, for women at this parity (Table 5).

The variance at the country-level was always highly statistically significant, which justifies the adoption of a multilevel approach. The set of country-level variables explained a substantial part of the variance at the country-level in all of the three models (Model I, II, and II) and for all of the three parities, as suggested by the decline in the country-level variance observed after the country-level variables had been included in the models.

Nevertheless, the only country-level variable with a statistically significant effect on women's lifetime fertility intentions was the share of highly educated women (Table 5). The statistical significance of this variable disappeared in the parity-two models. Although significance level should be interpreted with some caution, given that only 27 countries at level-two units are available, this result points out that the positive contextual effect of women's education on women's fertility intentions is mainly exerted at the beginning of the reproductive career (parity zero and one) and that the other country-level variables, childcare, gender, and GDP per capita, are not really working as mediator factors in the education-fertility intentions relationship.

To test whether the positive effect of women's education on intentions varied across countries, a random slope was also included in the models. These more sophisticated specifications did not, however, improve the fit of the model, which implies that being highly educated has the same effect on fertility intentions regardless of the country considered.^1^

The country share of women enrolled in education was initially also included in the set of the country-level covariates. The effect of this variable was positive, but only very weak and never statistically significant. Hence, it was not retained in the final models.

In addition, the positive contextual effect of education on intentions was not merely due to compositional effects: by comparing models with only country-level variables with those with both individual- and country-level variables, the magnitude and the sign of the coefficient related to the country share of highly educated women did not substantially change.

Importantly, the other country-level indicators included in the model with the aim to explain the cross-country variance in the women's fertility intentions, namely: pre-school children's participation in formal care, gender empowerment measure, and GDP per capita, did not change the magnitude or the significance level of the coefficient related to the share of women highly educated in the country. This implies that education at country-level does not simply capture the effects of some other close correlates, like gender equality, availability of childcare, or economic conditions in the country, as supposed in the research hypotheses.

## 6 Summary and discussion

The analysis, based on the 2006 and 2011 EB data, has revealed that the share of highly educated women in a European country is positively associated with women's lifetime fertility intentions. There is a positive contextual effect of women's education which has an impact on women's fertility intentions above and beyond that—also positive—of women's own level of education at the individual-level.

Unlike in developing countries, in Europe women who invest more resources in human capital do not necessarily plan to have fewer children than their less educated counterparts. This finding is in line with those of recent research on fertility (Esping-Andersen, 2009; Kravdal & Rindfuss, 2008). Results of the ordinal regression models on additionally intended number of children with a random intercept (Table 5) resemble those of empirical studies on higher-order actual fertility and, moreover, a good correspondence between intended and actual fertility has been found at the aggregate level, which does not necessarily imply a consistency also at the individual-level.

What could be the reason for this positive correlation between women's education and lifetime fertility intentions at individual- and country-level?

I hypothesised that this result might be explained by factors that increase the income effect and reduce the substitution effect of high levels of education among women in a given country: namely, access to childcare, gender equality, and good economic conditions. None of these a priori statements could be fully supported in the empirical analysis. Indeed, even after controlling for childcare, gender equality, and economic conditions at the country-level, the share of highly educated women appears as the only country-level variable with a statistically significant effect in the multilevel models.

These results seem to suggest that countries in which women are more likely to reach the highest educational levels are also the countries in which other structural circumstances encouraging fertility (that are not controlled for in this analysis) are more widespread, such as individuals’ sense of well-being, levels of trust (Aassve & Pessin, 2012), levels of happiness (Margolis & Myrskylä, 2011), or life satisfaction (Testa, 2012a).

The marriage market could also play an important role, given that highly educated women have a greater chance of marrying, a lower probability of divorcing, and a higher probability of having a partner who is better educated, and thus, more likely to plan to have larger families. Actually, the marriage market has been indicated as one of the reasons why school reforms which prolonged the time invested in education have had positive effects on fertility levels (Fort et al., 2011).

An intriguing explanation—which needs to be supported by empirical data—is related to feedback spill-over effects that the actual fertility of highly educated women might have on the intended fertility of highly educated women of younger reproductive ages: i.e., the more children highly educated women manage to have, the more children younger highly educated women who have not yet completed their reproductive careers will plan to have, because they see that it was possible for (presumably older generations of) women to combine both career and family. In other words, I assume that an increase over time in the share of highly educated women in the country will make successive generations of highly educated women more likely (to plan) to have larger families than their predecessors, who, as innovators of a new pattern of behaviour (i.e., the postponement of childbearing; see Billari & Philipov, 2004), faced many more challenges. The positive cross-country correlation between the share of highly educated women and their mean actual family size (Fig. 4) would be in line with this interpretation but it would remain unexplained why successive cohorts of women are more and more successful in achieving a large family size. Unfortunately, this issue cannot be investigated in more depth with the data at hand but it is certainly a fruitful line of research for future studies.

One should bear in mind that childbearing intentions depend not only on the individuals’ preference structure but also on country specific institutional contexts (Neyer, 2006). The countries in which the women with higher levels of education have more children might also be the countries in which policies introduced in past years have made it easier to combine work and family life, which might have had positive repercussions for fertility intentions of highly educated women. This is consistent with the similarity observed between the clustering of the countries according to the mean ultimately intended family size (Fig. 1) and the clustering of the countries according to the mix of policies in support to families introduced in the past (Thévenon, 2011).

The data have some limitations. First, they are cross-sectional and thus they do not allow a dynamic study of the fertility decision-making process. Second, the limited national sample sizes prevents any detailed and reliable analysis at the national level, and moreover, the limited information available at the individual level (the data do not, for example, contain any information on the partner's characteristics) may cause the results to be biased due to omitted relevant variables. Third, they do not allow a modelling of the selection effects generated by the postponement of childbearing among highly educated women. Being at an earlier stage of reproduction implies that highly educated women could still plan to have a greater number of children, and that their less educated counterparts observed at the same parity (i.e., the control group) can be selected out of the group for some unobserved characteristics, such as fecundity impairments, which may have a depressing effect on their stated lifetime fertility intentions. Moreover, the causal direction is assumed in this analysis to run from education to fertility, although in actually there will be some degree of reverse causation, especially in the case of educational enrolment (Cohen et al., 2011), which we are not able to model with the data at hand. Eventually, 27 countries are not enough to produce very robust and reliable estimates at the country-level, especially if several country-level variables are included in the models. Since the regional division of the EB data does not correspond to the NUTS 1 of the Eurostat, it was not possible to conduct the analysis at regional level while taking the regional-level variables from the statistics provided by Eurostat. It is hoped that it will be possible to address the issue in future studies on the basis of other data.

Nevertheless, the findings reported in the current study provide new insights into the women's fertility decision-making by bridging a link between macro-level factors and micro-level determinants of reproductive intentions. Building upon existing literature, they reveal that when it comes to women's lifetime fertility intentions education level has a positive effect both at the individual- and country-level. This means that the positive effects stemming from the higher degree of (perceived) behavioural control among highly educated women more than counterbalance the negative effects stemming from their increased level of autonomy and that these positive effects are reinforced in countries with a high share of highly educated women. Indeed, as seen in the analysis reported here, the women's preference structure is influenced by aggregate education; this means that low-educated women, who live in a society where the average educational level of women is high, have higher fertility intentions than if they live elsewhere. Although nothing was learned about the underlying mechanism, education at contextual level deserves attention in future research on fertility in Europe.

The results are rich in implications for policy makers. The increased investments in education may have positive effects on fertility levels if the obstacles that prevent highly educated women from combining family life with a career appropriate to their human capital are removed through adequate policy measures. As education tends to be passed on from one generation to the next, these policy interventions will ultimately increase a country's human capital resources, and thus its productivity, not just today, but into the future.

## Figures and Tables

**Table 1:** Structure of the data: women aged 20 to 45 by country and parity EB 2006 and EB 2011 pooled dataset.

Countries	Parity		
	0	1	2
Austria	189	117	135
Belgium	137	83	136
Bulgaria	93	127	146
Cyprus	74	27	54
Czech Republic	125	132	216
Denmark	116	59	103
Estonia	83	120	134
Finland	103	68	97
France	112	99	148
Germany	181	131	175
Greece	188	78	145
Hungary	101	103	153
Ireland	106	87	114
Italy	237	121	135
Latvia	126	158	173
Lithuania	118	108	134
Luxembourg	60	42	83
Malta	49	36	72
Netherlands	135	58	135
Poland	112	91	102
Portugal	95	107	108
Romania	131	151	116
Slovakia	140	131	177
Slovenia	166	98	116
Spain	137	103	149
Sweden	74	53	95
UK	144	139	142

Total	3332	2627	3493

**Table 2:** Description of the individual- and country-level variables used in the analysis. EB 2006 and 2011 pooled dataset. Women aged 20–45.

	Parity		
	0	1	2
*(a) Individual-level variables*. Percentage distributions			
Age (average)	29	34	37
Year 2011	48	49	46
Year 2006	52	51	54
Married	17	60	75
Cohabiting	25	16	10
Single	52	11	4
Separated	6	12	11
Low education	5	9	12
Medium education	34	54	54
High education	38	36	33
Enrolled in education	23	2	1
Employed	62	66	68
Unemployed	10	14	10
Inactive	27	20	22
Low self-positioning on the social scale^a^	54	60	59
High self-positioning on the social scale	46	40	41

	GDP per capita (in pps)		Gender empowerment measure		Pre-school children in formal childcare services (%)		Women with high level of education (%)	
	2006	2011	2006	2008	2006	2010	2005	2008
*(b) Country-level variables*								
Austria	126	131	0.815	0.744	75	78	33	32
Belgium	118	119	0.855	0.874	99	99	45	43
Bulgaria	38	47	0.595	0.613	75	71	28	26
Cyprus	93	91	0.584	0.603	85	73	48	49
Czech Republic	80	79	0.615	0.664	82	79	15	18
Denmark	124	125	0.861	0.896	91	94	37	37
Estonia	66	69	0.608	0.665	85	90	52	46
Finland	114	115	0.853	0.902	48	56	45	43
France	108	108	0.718	0.779	100	101	36	38
Germany East	116	122	0.816	0.852	89	94	33	32
Greece	92	75	0.614	0.677	47	48	39	40
Hungary	63	66	0.560	0.590	87	87	21	25
Ireland	146	130	0.753	0.722	49	79	52	53
Italy	105	99	0.653	0.741	99	97	17	21
Latvia	53	62	0.621	0.648	77	81	38	36
Lithuania	58	70	0.635	0.628	61	66	55	56
Luxembourg	271	272	0.653	0.653	86	87	34	34
Malta	79	86	0.493	0.531	91	94	27	27
Netherlands	131	129	0.844	0.882	58	67	33	35
Poland	52	66	0.610	0.631	41	60	28	33
Portugal	79	75	0.681	0.753	79	84	19	23
Romania	38	49	0.492	0.512	73	73	16	18
Slovakia	63	75	0.599	0.663	73	72	12	16
Slovenia	88	82	0.603	0.641	78	86	26	28
Spain	105	97	0.776	0.835	98	99	37	39
Sweden	123	129	0.883	0.909	86	93	46	44
UK	121	110	0.755	0.790	91	93	32	34

a Respondents were asked to position themselves on the social scale. The scale had 10 levels: one for the lowest level in society and ten for the highest level in society. Sensitivity analysis based on different coding of the variables also as numerical variable rather than dummy variable has suggested that the latter captures the variation in the answers at best. This variable is not available in the 2006 Eurobarometer round.

**Table 3:** Distribution of women aged 20–45 by actual and ultimately intended family size. EB 2011.

	Actual family size			U-shape	Ultimately intended family size			U-shape
	0	1	2		0	1	2	
*Panel (a) high-educated women*								
Austria	38	26	36	×	4	37	58	
Belgium	35	19	46	×	11	13	77	
Bulgaria	30	38	33		0	19	81	
Cyprus	59	11	30	×	5	7	89	
Czech Rep.	25	35	41		4	11	85	
Denmark	21	25	54	×	8	10	82	
Estonia	23	33	44		1	9	90	
Finland	30	18	52	×	10	11	79	
France	32	24	44	×	2	13	85	
Germany West	26	23	51	×	7	21	72	
Germany East	29	32	39		7	25	68	
Greece	54	17	29	×	5	21	74	
Hungary	38	23	39	×	0	19	81	
Ireland	25	29	46		3	7	90	
Italy	36	29	35	×	6	16	78	
Latvia	26	35	39		5	17	77	
Lithuania	33	25	42	×	1	15	84	
Luxembourg	36	20	45	×	9	11	81	
Malta	48	16	36	×	5	19	76	
Netherlands	45	14	41	×	14	8	78	×
Poland	31	32	37		2	21	77	
Portugal	50	21	29	×	11	27	62	
Romania	41	46	12		2	36	62	
Slovakia	39	23	38	×	2	16	82	
Slovenia	32	32	36		4	17	80	
Spain	37	21	42	×	3	17	80	
Sweden	30	26	44	×	3	10	87	
U. Kingdom	33	28	39	×	9	4	87	×

Total number of countries				19				2

	Actual family size			U-shape	Ultimately intended family size			U-shape
	0	1	2		0	1	2	
*Panel (b) medium and low-educated women*								
Austria	39	20	42	×	13	21	67	
Belgium	21	23	56		9	16	75	
Bulgaria	18	34	48		2	25	73	
Cyprus	16	20	64		0	20	80	
Czech Republic	21	25	54		1	17	82	
Denmark	37	15	48	×	9	0	91	
Estonia	15	28	57		2	14	84	
Finland	23	20	57	×	4	18	78	
France	15	24	61		4	10	85	
Germany West	28	18	54	×	12	15	73	
Germany East	30	30	39		14	20	66	
Greece	26	22	52	×	3	16	81	
Hungary	19	30	51		7	19	73	
Ireland	21	23	56		8	7	85	×
Italy	39	26	35	×	9	23	68	
Latvia	11	37	52		2	22	76	
Lithuania	23	27	49		3	17	80	
Luxembourg	17	23	60		11	15	74	
Malta	26	22	52	×	5	26	70	
Netherlands	33	16	51	×	11	12	77	
Poland	15	29	56		2	19	79	
Portugal	17	34	49		5	28	66	
Romania	13	36	51		4	33	63	
Slovakia	24	27	49		2	25	73	
Slovenia	14	31	56		2	22	75	
Spain	19	31	50		5	16	79	
Sweden	12	28	60		7	23	70	
United Kingdom	21	29	50		10	15	75	

Total number of countries				8				1

*Note*: The row percentages sum up to 100 in each panel. The countries with a U-shape distribution are those in which the proportion of women with only one child (or only one ultimately intended child) is lower than the proportions of women with zero and two children (actual or ultimately intended). Women at parity three or above have been excluded from this analysis.

**Table 4:** Mean actual, mean additionally intended and mean ultimately intended family size by level of education.^a^ EB 2011.

	Actual family size (AFS)		Additionally intended family size (AIFS)		Ultimately intended family size (UIFS)		Countries in which highly educated women have a mean UIFS bigger, equal, or smaller than the less educated counterparts		
	Low edu	High edu	Low edu	High edu	Low edu	High edu	High > low	High = low	High < low
Austria	1.2	1.1	0.6	0.4	1.8	1.5			×
Belgium	1.6	1.4	0.5	0.8	2.1	2.2	×		
Bulgaria	1.4	1.0	0.5	0.9	1.9	1.8			×
Cyprus	1.9	0.9	0.5	1.3	2.4	2.1			×
Czech Rep.	1.5	1.2	0.6	0.8	2.1	2.0			×
Denmark	1.5	1.6	1.3	0.6	2.8	2.2			×
Estonia	1.7	1.4	0.6	1.0	2.3	2.4	×		
Finland	1.8	1.5	0.7	0.8	2.5	2.3			×
France	1.9	1.3	0.7	1.1	2.6	2.4			×
Germany	1.5	1.3	0.5	0.8	2.0	2.0		×	
Greece	1.4	0.9	0.7	1.1	2.1	2.0			×
Hungary	1.6	1.2	0.7	1.0	2.3	2.2			×
Ireland	1.9	1.7	0.8	1.2	2.7	2.9	×		
Italy	1.0	1.1	0.8	1.1	1.8	2.2	×		
Latvia	1.7	1.2	0.5	0.8	2.2	2.0			×
Lithuania	1.5	1.2	0.7	1.0	2.2	2.2		×	
Luxembourg	1.8	1.3	0.3	0.7	2.1	2.0			×
Malta	1.4	1.1	0.5	1.1	1.9	2.2	×		
Netherlands	1.4	1.0	0.8	0.9	2.1	1.9			×
Poland	1.7	1.2	0.5	1.0	2.2	2.2		×	
Portugal	1.5	0.8	0.4	0.9	1.9	1.7			×
Romania	1.6	0.7	0.2	1.0	1.8	1.7			×
Slovakia	1.4	1.0	0.6	1.4	2.0	2.4	×		
Slovenia	1.6	1.2	0.5	0.9	2.1	2.1		×	
Spain	1.3	1.1	0.5	0.8	1.9	1.9		×	
Sweden	1.8	1.4	0.3	1.0	2.1	2.4	×		
United Kingdom	1.7	1.2	0.7	1.0	2.4	2.2			×

No of countries					7	5	15		

a Low education category includes low- and medium-educated women.

**Table 5:** Estimates from ordinal multilevel regression models on the additionally intended number of children. Beta coefficients.

Models	Parity 0			Parity 1			Parity 2		
	I	II	II	I	II	III	I	II	III
*Individual-level variables*									
Age-33 (average)	–	−0.22***	−0.22***	–	−0.21***	−0.21***	–	−0.16***	−0.16***
(Age-33)^2	–	−0.01***	−0.01***	–	−0.01***	−0.01***	–	−0.003*	−0.003*

Year 2011 (reference)	–	0	0	–	0	0	–	0	0
Year 2006	–	−0.04	−0.04	–	0.13	0.20*	–	−0.11	−0.12

Married (reference)	–	0	0	–	0	0	–	0	0
Cohabiting	–	0.01	0.01	–	0.19	0.17	–	0.09	0.07
Single	–	−0.00	−0.00	–	−0.44**	−0.47**	–	0.20	0.19
Separated	–	−0.34	−0.34	–	−0.62***	−0.63***	–	0.41	0.41

Low education (reference)	–	0	0	–	0	0	–	0	0
Medium education	–	−0.06	−0.07	–	0.25	0.25	–	−0.02	−0.03
High education	–	0.34*	0.33+	–	0.79***	0.78***	–	0.55**	0.51*
Enrolled in education	–	0.72**	0.73**	–	1.25***	1.22***	–	1.80	0.73

Employed (reference)	–	0	0	–	0	0	–	0	0
Unemployed	–	0.02***	0.01***	–	0.07	0.08	–	0.16	0.18
Not participating in the labour force		−0.43*	−0.43*		0.11	0.12		0.04	0.03

Low pos. on the social scale (reference)	–	0	0	–	0	0	–	0	0
High positioning on the social scale	–	0.19**	0.19**	–	0.42***	0.41***	–	0.37**	0.36**

*Country-level variables*									
Women with high level of education (%)	–	–	0.02**	–	–	0.01+	–	–	0.01
Pre-school children in formal childcare (%)	–	–	0.002	–	–	0.01	–	–	−0.01
Gender Empowerment Measure	–	–	−0.85	–	–	1.35	–	–	0.58
Log GDP per capita	–	–	0.03	–	–	−0.18	–	–	0.21

First cut-point	−1.42***	−1.06***	−0.66	−0.03	0.19	1.35	1.87***	1.85***	3.03***
Second cut-point	−0.60***	0.11	0.50	1.82***	2.65***	3.81***	2.81***	2.90***	4.08***
Third cut-point	1.57***	2.65***	3.05***	3.85***	4.85***	6.01***	4.64***	4.77***	5.95***

Variance at the country-level	0.19***	0.15***	0.11***	0.18***	0.16***	0.11***	0.25***	0.19***	0.15***

Level-one units: individuals	3332	3332	3332	2627	2627	2627	3493	3493	3493
Level-two units: countries	27	27	27	27	27	27	27	27	27

* p <. 05.

** p < .01.

*** p < .001

## Footnotes

1 This result has been supported by additional analysis carried out separately on each single EU country (results are not shown in the paper but available upon request).
